# COVID‐19 Vaccine Uptake and Socioeconomic Disparities in Tanzania: A Population‐Based Cross‐Sectional Study Amid High Hesitancy

**DOI:** 10.1002/hsr2.71044

**Published:** 2025-07-11

**Authors:** Deogratius Bintabara, Gabriel Mchonde

**Affiliations:** ^1^ Department of Community Medicine The University of Dodoma Dodoma Tanzania; ^2^ Department of Anatomy and Histology, School of Medicine and Dentistry The University of Dodoma Dodoma Tanzania

**Keywords:** COVID‐19 vaccine, education, regional disparities, socioeconomic disparities, Tanzania, vaccine hesitancy

## Abstract

**Background and Aims:**

Socioeconomic disparities in healthcare access, including vaccination services, persist worldwide. The COVID‐19 pandemic amplified these disparities, particularly in low‐resource settings such as Tanzania, where vaccine hesitancy remains a significant challenge. Understanding these disparities is crucial for achieving equitable vaccine coverage. This study assessed regional variations in socioeconomic disparities in COVID‐19 vaccine uptake in Tanzania, with education used as a proxy for socioeconomic status. The findings aim to guide targeted interventions for populations facing greater disadvantages.

**Methods:**

A population‐based cross‐sectional survey was conducted between June and July 2022, involving 22,910 adults from urban areas in Mwanza, Iringa, Arusha, and Morogoro regions. Participants were selected using cluster sampling. Vaccine uptake, defined as receiving any complete dose of the COVID‐19 vaccine, was the primary outcome. Disparities were analyzed using concentration curves and indices, while multivariable logistic regression identified factors contributing to these disparities.

**Results:**

Of the respondents, approximately 20% were fully vaccinated. Significant educational disparities favoring the educated were observed, with the most pronounced gaps in Mwanza (CI: 0.093, *p* < 0.001) and Arusha (CI: 0.062, *p* < 0.001) compared to Iringa (CI: 0.011, *p* < 0.01) and Morogoro (CI: 0.040, *p* < 0.001). Multivariable analysis identified age, marital status, occupation, and knowledge as potential factors associated with vaccine uptake disparities. Educated individuals were significantly more likely to be vaccinated, underscoring the role of education in shaping vaccine access and acceptance.

**Conclusions:**

This study highlights the need to address regional and socioeconomic disparities in COVID‐19 vaccine uptake in Tanzania. Tailored policies that consider education levels, employment type, and community perceptions are essential to improving vaccine coverage. Efforts must focus on reducing hesitancy and promoting equitable access, particularly among disadvantaged groups.

## 1 Background

Emerging infectious diseases (EIDs) have been linked to significant socioeconomic and political consequences, often exacerbating inequalities within and among human societies. These diseases may arise from novel viruses causing global outbreaks with severe impacts on public health. As of March 2024, the World Health Organization (WHO) reported that over 774 million people worldwide have been infected with coronavirus disease‐2019 (popularly known as COVID‐19), with more than 7 million deaths attributed to the virus [1]. To curb the spread of such diseases, current preventive and control strategies include early screening, isolation, and treatment [2].

Vaccinating a whole population based on the recommendations of health authorities and professionals around the world has been given high priority to limit the spread of the disease. This strategy has been considered most important in public health as it has prevented the spread of dangerous infectious diseases efficiently and effectively [3, 4]. Even though about 13.5 billion vaccine doses have been administered globally, only around 5.55 billion (72.3%) and 5.1 billion (66.2%) people of the world population have received at least one or complete dose of the COVID‐19 vaccine, respectively. However, of all WHO regions, Africa seems to have a low rate of vaccination, in which only 32.6% of its population is fully vaccinated [1, 5].

In Tanzania, the COVID‐19 vaccination campaign commenced in July 2021, marked by President Samia Suluhu Hassan publicly receiving the Johnson & Johnson vaccine, signaling a significant shift in the national approach [6]. The initial rollout prioritized healthcare workers and vulnerable populations. Tanzania received its first batch of over 1 million Johnson & Johnson vaccine doses through the COVAX initiative, donated by the United States [7]. Additional vaccines such as Pfizer‐BioNTech, Sinopharm, and Sinovac were later introduced through both COVAX and bilateral agreements [8]. Despite these efforts, vaccine uptake in Tanzania faced multiple challenges or factors including vaccine hesitancy, limited access in rural areas, and disparities based on socioeconomic status (SES) [9, 10]. Urban residents typically had more consistent access to vaccination services, whereas rural and low‐income communities encountered logistical and informational barriers [11, 12]. To improve coverage, the government integrated COVID‐19 vaccination into routine immunization and primary health services to reach underserved populations [13].

Hesitance has been reported as among the major reasons for the low rate of COVID‐19 vaccination in most African countries. However, Tanzania is among the few countries in this WHO region performing better, but still, there is high hesitancy against the COVID‐19 vaccine in some of its regions [14]. Also, some studies indicated that SES, such as income/wealth, occupation, and education, plays an important role in determining health outcomes such as uptake of the COVID‐19 vaccine [15]. Literature documented that having low SES means being poor, unemployed, or less educated correlated with a higher risk of poor health outcomes such as unhealthy behavior, ill health, mortalities, and difficulties in accessing healthcare such as vaccination services [16, 17]. A previous study conducted in Tanzania used wealth to assess health‐related inequalities and found that as household wealth increased, women with at least primary education had significantly higher odds of utilizing key maternal health services [18]. In contrast, a study conducted in Mongolia used education as the primary measure of inequality and found that poor self‐rated health, physical limitations, and chronic illness were significantly more concentrated among less educated individuals [17]. These findings illustrate how different dimensions of SES such as wealth in Tanzania and education in Mongolia have been instrumental in assessing inequalities in both access to and utilization of health services, as well as in evaluating health outcomes [16, 17, 18, 19]. However, little is known regarding to what extent there are socioeconomic inequalities in accessing the COVID‐19 vaccine in Tanzania.

Therefore, examining socioeconomic inequalities in accessing the COVID‐19 vaccine in Tanzania is of greater importance. In this study, education‐related inequality was used as a proxy indicator of socioeconomic inequalities in accessing the COVID‐19 vaccine. Education is often preferred in assessing socioeconomic inequalities because it is stable, easy to measure, and is most used as a proxy indicator of income, status, or social class [20, 21, 22]. Also, it is less prone to nonresponse error than income or wealth as a socioeconomic position indicator [23]. This study, therefore, was aimed at assessing the regional variation of socioeconomic‐related inequalities in the uptake COVID‐19 vaccine in Tanzania.

## 2 Materials and Methods

### 2.1 Study Setting

This survey was conducted in the urban residential neighborhoods across the major regions (cities) in Tanzania. The selected urban cities are Mwanza, Iringa, Arusha, and Morogoro regions. These urban sites were selected because are among the sites in which the incidence of SARS‐CoV‐2 infections has been highly reported compared to rural sites. Furthermore, reliable community data are absent due to limited access to testing capacities and undetected mild courses or asymptomatic infections, in those who have been exposed to the SARS‐CoV‐2 virus.

### 2.2 Study Design and Population

A population‐based cross‐sectional survey was conducted between June and July 2022 that interviewed adult men and women residents of selected cities, who were eligible to participate in the study.

### 2.3 Sample Size and Sampling Technique

The sample size for this study was estimated using a cluster sampling formula based on guidelines provided by the United Nations Department of Economic and Social Affairs (UN DESA) for household sample surveys in developing countries [24]. The estimated sample size for each region was 5810 adults, calculated by using the following assumption: an expected vaccination coverage of 50%, level of significance at 95%, marginal error of 2%, a design effect of 2.2 to account for clustering, and an additional 10% to compensate for potential nonresponse rate. Therefore, the total sample for all four regions was estimated to be 23,240.

The one‐stage cluster sampling technique was used to obtain the study participants in the selected urban district/council in these four regions. From each selected district/council, a sampling frame that consists of all wards was developed. Thereafter, a total of 10 wards were chosen from the sampling frame by using a simple random sampling technique. Finally, all households within the selected wards were visited sequentially, and adults aged 18 years or older who were usual residents and provided informed consent were recruited for the study. Individuals were excluded if they were temporary visitors to the household, unable to respond to the interview due to cognitive or physical impairments, or declined to participate. In households with more than one eligible adult, only one was randomly selected by using the lottery method.

### 2.4 Data Collection and Management

The interviewer‐administered questionnaires were used during data collection aiming at involving both literate and illiterate participants. First, the expert (professional translator) translated the English version into Swahili language. Thereafter, another professional translator did a back translation to English to check for its original meaning. The final version was pretested in the Dodoma region (this region was not included in the main survey). A total of 100 third‐year medical students (MD3) were trained for 5 days from June 20 to June 24, 2022, and participated in the pretesting exercise. The actual data collection took place from June 27 to July 10, 2022, with interviews conducted under the close supportive supervision of the principal investigator and eight coinvestigators. To ensure data quality, at the end of each day, all questionnaires were counterchecked for completeness and consistency of the responses before leaving the field site. For all questionnaires that were not filled properly, we identified the interviewers and returned to the respective household and filled the information correctly.

### 2.5 Measurement of Variables

#### 2.5.1 Outcome Variable

Uptake of COVID‐19 vaccination. This was categorized as “Yes” if an adult reported having been vaccinated by any complete dose against SARS‐CoV‐2 viruses; otherwise, categorized as “No.”

#### 2.5.2 Independent Variables

The independent variables measured in this study included a range of sociodemographic, health‐related characteristics, and knowledge, attitude, and perception toward COVID‐19 vaccination. The region of residence was categorized into four areas: Arusha, Iringa, Morogoro, and Mwanza. Age was treated as a continuous variable with a median of 32 years and an interquartile range (IQR) of 25–45 years; for analytical purposes, it was further categorized into four groups: < 20 years, 20–34 years, 35–49 years, and ≥ 50 years. Sex was recorded as either male or female. Marital status was grouped into two categories: living with a partner (married or cohabiting) and single, widowed, or divorced. Education level was classified as none, primary, secondary, or tertiary. In this study, *none* referred to participants who had not attended formal education; *primary* included those who had completed Standards 1–7; *secondary* included those who had attended Forms 1–4 and/or Forms 5–6; while *tertiary* referred to individuals who had attended higher education, such as college or university. Participants' occupation status was categorized into employed in the formal sector, self‐employed, petty trader, housewife, student, or other activities. Additionally, participants were asked whether they had any chronic diseases, with responses categorized as yes or no.

Participants' knowledge regarding COVID‐19 vaccines was assessed using eight question items, each with three possible responses: “Yes,” “No,” and “Don't know.” A score of 1 was assigned for each item answered with “Yes,” indicating agreement or correct knowledge, while a score of 0 was given for other responses. The total knowledge score was calculated by summing the scores from all eight items. Participants whose total score was above the overall mean were classified as having *good knowledge*, whereas those scoring at or below the mean were classified as having *poor knowledge*.

Attitude toward COVID‐19 vaccines was evaluated using nine items measured on a three‐point Likert scale: “Agree,” “Disagree,” and “Neither agree nor disagree.” Participants received a score of 1 for each item they responded with “Agree” and 0 for other responses. The total attitude score was computed by summing the responses to all nine items. Participants with scores above the mean were categorized as having a *positive attitude*, whereas those scoring below or equal to the mean were considered to have a *negative attitude*.

Perceptions related to COVID‐19 vaccines were assessed through five items using the same three‐point Likert scale. As with the previous scales, a score of 1 was given for “Agree” responses and 0 for all others. The total perception score was the sum of the five items. Participants with scores above the mean were considered to have a *positive perception*, whereas those with scores at or below the mean were categorized as having a *negative perception*.

The items used to assess and scoring knowledge, attitude, and perception as well as the scoring and categorization approaches were adapted from validated tools previously applied in similar studies on COVID‐19 vaccination in SSA countries [25, 26, 27].

### 2.6 Statistical Analysis

The descriptive analysis was performed, in which all continuous variables were summarized using median and IQR, whereas categorical variables were summarized by using proportions and then presented as tables and graphs. An unadjusted logistic regression model was employed to assess any association between the outcome (uptake of COVID‐19 vaccine) and independent variables. Then, all variables with a *p*‐value less than 0.2 were considered for inclusion into the multivariable logistic regression (Adjusted) model using the stepwise (backward elimination) method to test for the association of each variable with the outcome variable by adjusting for all variables included in the model. The *p*‐value < 0.05 and 95% confidence interval (CI) for the odds ratio (OR) were used to confirm the significance of the associations. Finally, all adjusted odds ratios (AORs) and their 95% CI were graphically presented using a forest plot. In this plot, the measure of association (AOR) with the corresponding CI is represented by a bolded dot with horizontal lines.

In addition, the following two methods were used to evaluate inequalities in the uptake of COVID‐19 vaccination, that is, construction of concentration curves (CCs) and the computation of concentration indices (*C*).

#### 2.6.1 Concentration Curves

The CC was used to evaluate the patterns of inequalities in the uptake of COVID‐19 vaccine. The CC plots the cumulative percentage of the outcome variable (*y*‐axis) against the cumulative percentage of the population ranked by education level, beginning with the lowest level (None), and ending with the highest level (*x*‐axis). In other words, they plot shares of the outcome variable against quintiles of the education levels.

#### 2.6.2 Concentration Indices (*C*)

The computation of *C* has an additional advantage compared to CC for quantifying the degree of educational‐related inequality in healthcare variables such as the uptake of COVID‐19 vaccination. This *C* is defined as “twice the area between CC and the line of equality.” It takes the values bounded between −1 and +1. If the health variable is “good” such as uptake of vaccination, an index with a positive value indicates uptake of vaccination is more among the educated [28, 29]. Mathematically, the *C* can be calculated by using the following Formula (1).

(1) $$C=\frac{2}{\mu }\;\mathrm{cov}(y,r)$$

Because the outcome variable is binary, the bounds of the *C* may not lie between −1 and +1. The two techniques that produce Wagstaff and Erreygers *C* are frequently used to account for the aforementioned limitation [30, 31]. Therefore, this study used the Erreygers *C* which satisfies four properties of the rank‐dependent measure of inequality [32, 33]. All statistical analyses were performed using STATA version 17.

### 2.7 Ethical Considerations

This study took care not to infringe on ethical issues. Therefore, Ethical clearance was requested and finally approved by The University of Dodoma (UDOM) Research Ethics Committee (reference MA.84/261/02). The respondents were adequately informed using the participant's informed consent statement about all the relevant aspects of the study, including its aim, interview procedures, anticipated benefits, and potential hazards. Also were informed that participating in the study will be entirely voluntary; therefore, they have the right to participate or to abstain from participation and can terminate their participation at any time, whenever they feel that they do not want to continue. In this case, the respondents become subjects of the study if agree and provide signed written informed consent or thumbprint for those who are not able to write. To maintain confidentiality, interviews were done in privacy; no names of participants were recorded in the data collection form. Therefore, this study was conducted following the guidelines and regulations of the Declaration of Helsinki.

## 3 Results

### 3.1 Baseline Characteristics

A total of 22,910 participants were included in the analysis, drawn from four regions of Tanzania. As shown in Table 1, the median age of the respondents was 32 years (IQR: 25–45). The majority (57%) of the respondents were living with their partners at the time of the interview. About 10% of respondents reported having formal employment while approximately 8% had not attended any formal education. In addition, about 11% reported having known chronic diseases such as diabetes or hypertension.

**Table 1:** Distribution of baseline characteristics among participants of this study (*n* = 22,910).

Variable	*n* (%)
Region	
Arusha	5709 (24.92)
Iringa	6130 (26.76)
Morogoro	5840 (25.49)
Mwanza	5231 (22.83)
Age (median [IQR] = 32 [25–45])	
< 20	1089 (4.75)
20–34	11,475 (50.09)
35–49	6032 (26.33)
≥ 50	4314 (18.83)
Sex	
Male	10,791 (47.10)
Female	12,119 (52.90)
Marital status	
Living with partner	12,969 (56.61)
Single/widow/divorced	9941 (43.39)
Education level	
None	1779 (7.77)
Primary	3439 (15.01)
Secondary	8330 (36.36)
Tertiary	9362 (40.86)
Occupation status	
Employed in the formal sector	2400 (10.48)
Self‐employed	6175 (26.95)
Petty trader	6654 (29.05)
Housewife	2310 (10.08)
Students	2052 (8.96)
Other activities	1412 (6.16)
Any chronic diseases	
Yes	2683 (11.71)
No	20,227 (88.29)

### 3.2 Uptake of COVID‐19 Vaccine

Of the 22,910 adults who participated in the study, 4326 (18.9%) were reported to be fully vaccinated against COVID‐19. This indicates that less than one‐fifth of the study population had completed the recommended COVID‐19 vaccination schedule at the time of data collection, highlighting a relatively low uptake rate despite national efforts to promote vaccine coverage.

### 3.3 Factors Contributed to Socioeconomic Inequalities in Uptake of COVID‐19 Vaccine

**Figure 1:**
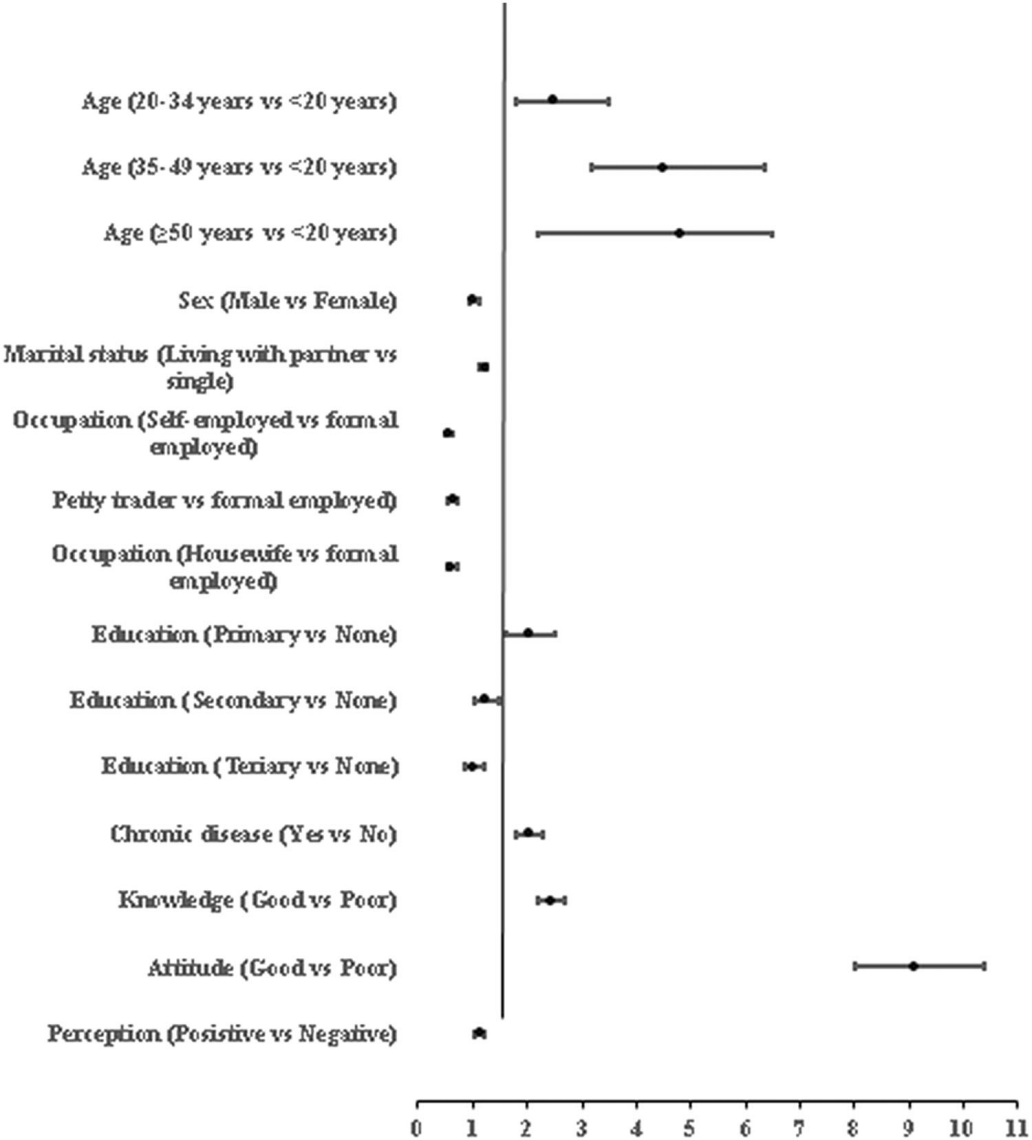
A forest plot of the adjusted logistic regression with the uptake of COVID‐19 vaccines as the dependent variable (*n* = 22,910).

Figure 1 presents the AORs with a 95% CI from the multivariable logistic regression model. Each variable was adjusted by all other variables included in the model. It is found that the likelihood of uptake COVID‐19 vaccine was always higher among educated adults (primary [AOR = 2.0; 95% CI: 1.63–2.49] and secondary [AOR = 1.2; 95% CI: 1.01–1.47]) compared to uneducated ones. In addition, the findings revealed that having good knowledge (AOR = 2.4; 95% CI: 2.21–2.69), good attitude (AOR = 9.1; 95% CI: 8.00–10.38), and positive perception (AOR = 1.1; 95% CI: 1.03–1.23) were significantly associated with uptake of COVID‐19 vaccines. Furthermore, the odds of uptake of COVID‐19 vaccines were higher among older age, those living with partners, those employed in the formal sector, and those with chronic disease.

**Figure 2:**
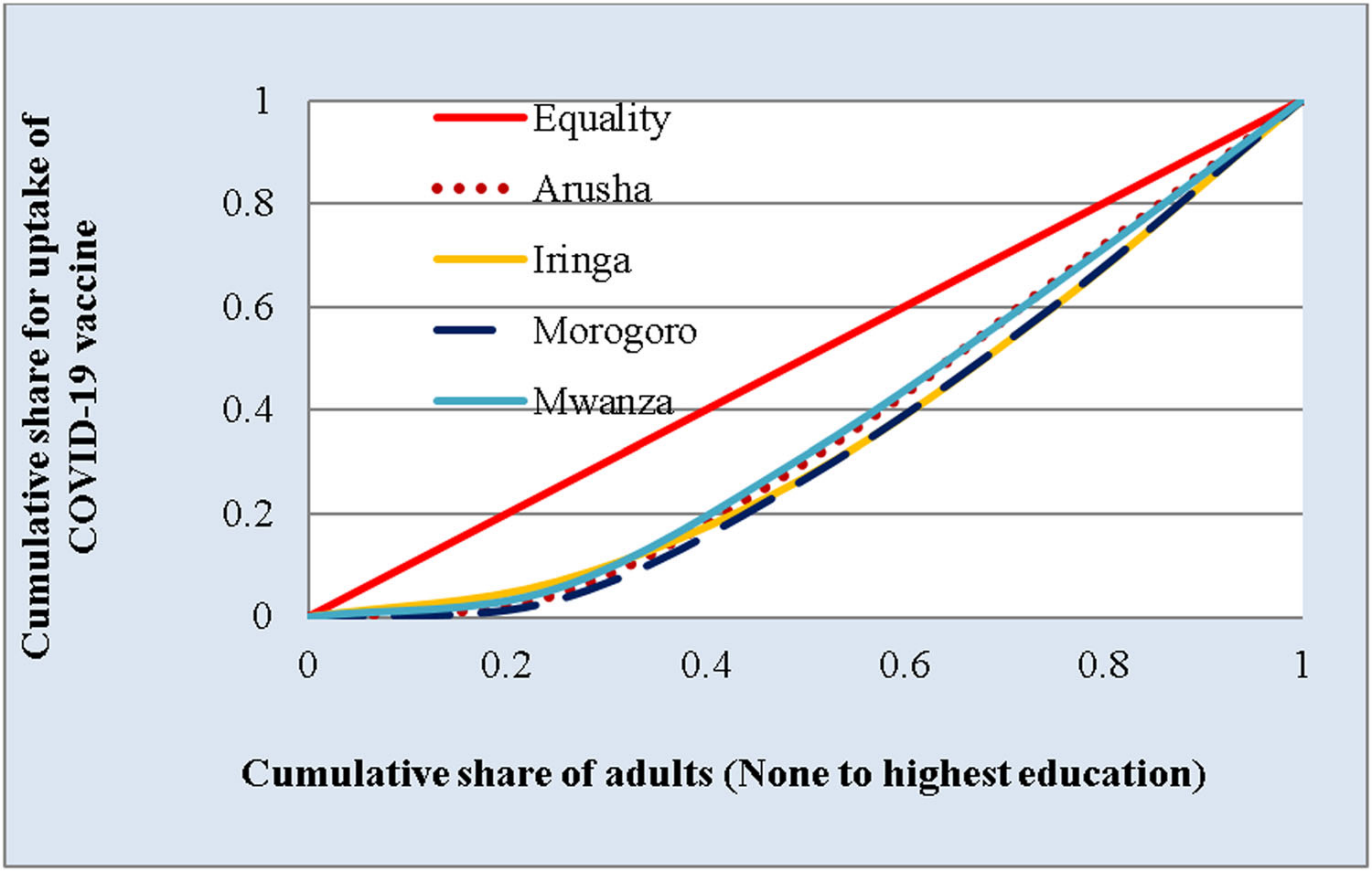
Concentration curves (CCs) for uptake of COVID‐19 vaccine by regions.

Figure 2 presents the CCs that were used to assess the presence of inequalities in accessing COVID‐19 vaccines across the selected regions in Tanzania. In all regions, the CCs are below the line of equality suggesting inequalities in accessing the COVID‐19 vaccine in favor of educated adults.

**Figure 3:**
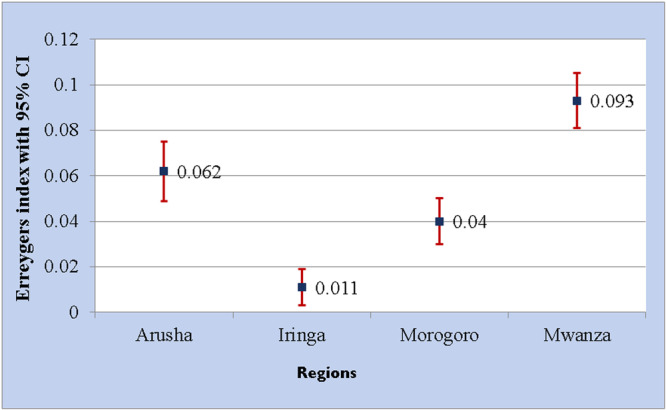
Regional variation of socioeconomic‐related inequality in uptake of COVID‐19 vaccine.

Figure 3 presents the estimates of the Erreygers's index (EI) with 95% CIs to summarize and compare the extent of education‐related inequality in the uptake of the COVID‐19 vaccine across the selected four regions in Tanzania. All the indices were found to be positive and statistically significant, indicating inequalities in the uptake of the COVID‐19 vaccine in favor of educated adults. Furthermore, the findings revealed significant regional variation in education‐related inequality in the uptake of the COVID‐19 vaccine. It shows the high extent of inequalities observed in Mwanza and Arusha compared to the Iringa and Morogoro regions.

## 4 Discussion

The current study is the first to examine the regional variation of socioeconomic inequalities in the uptake of the COVID‐19 vaccine in a country that never had mandatory lockdown during all waves of the COVID‐19 pandemic. Education‐related inequalities were used as a proxy measure to assess socioeconomic inequalities in the uptake of the COVID‐19 vaccine. The findings revealed that for all four selected regions in Tanzania, there was a significant socioeconomic inequality in the uptake of the COVID‐19 vaccine in favor of educating individuals. This suggests that educated adults were generally advantaged in the uptake of the COVID‐19 vaccine compared to uneducated. This is probably due to that educated adults are more knowledgeable and well understand the importance of primary preventive measures compared to uneducated adults. Likewise, the higher concentration of COVID‐19 vaccine uptake among educated individuals could be explained by the fact that education exposes someone to high social status or being in the wealthier group [34]. This situation helps them afford to purchase goods and services including paying the cost related to accessing primary preventive services such as vaccination services [35].

Furthermore, the observed socioeconomic inequalities in the uptake of the COVID‐19 vaccine suggest that the effort of introducing mass campaigns and regular door‐to‐door sensitization programs in Tanzania did not eliminate differences between educated and uneducated individuals in the uptake of the COVID‐19 vaccine. However, on the other hand, this inequality might be due to low vaccine acceptance among uneducated individuals compared to educated ones as documented in previous studies [36, 37]. Also, it has been reported that individuals with low SES such as those being uneducated were more likely affected by online vaccine misinformation during the pandemic resulting in their low acceptance rate toward the COVID‐19 vaccine [38]. This might explain the observed low rate of COVID‐19 vaccine uptake among uneducated individuals compared to educated. Furthermore, the findings from concentration indices and CCs highlighted the significant socioeconomic inequalities in the uptake of COVID‐19 vaccines in Tanzania. The estimates indicate that these inequalities were in favor of educated adults meaning that education probably has a greater impact on the reduction or elimination of inequalities in the uptake of COVID‐19 vaccines in Tanzania.

In addition, this study used multivariable analysis to identify the potential contributors to the observed socioeconomic inequalities in the uptake of the COVID‐19 vaccine. The analysis was able to unveil that age, marital status, occupation, chronic disease, knowledge, attitude, and perception as potential contributors to socioeconomic inequalities in the uptake of the COVID‐19 vaccine.

Our findings revealed that the uptake of COVID‐19 vaccination tends to increase among older ages compared to younger ages. This was also reported in a previous study conducted in similar settings [39]. Also, adults with chronic diseases such as hypertension and diabetes mellitus were more likely to uptake the COVID‐19 vaccination compared to their counterparts. The influence of these two factors (older age and having chronic diseases) can be explained due to that, they were important priority groups for COVID‐19 vaccination in developing countries such as Tanzania.

The current study also highlighted the significant influence of having any level of formal education on the uptake of the COVID‐19 vaccine. Similar findings have been reported in several previous studies [40, 41]. These findings could be explained by the fact that having any level of education is often associated with lower hesitancy and greater acceptance of vaccination uptake [42, 43]. Formal education can also increase access to information and trust in public health interventions. Furthermore, individuals with formal education are more likely to be employed in the formal sectors, which was among the priority groups for COVID‐19 vaccination during the rollout in Tanzania [40]. This can be evidenced in our findings which indicated that adults who have been employed in the formal sector were more likely to be vaccinated compared to their counterparts. The possible explanation for this finding is that those employed in the formal sector may require a vaccination certificate to access important services such as local and international travel for professional workshops or international conferences. Therefore, the rate of COVID‐19 vaccine might be higher in this group.

Moreover, the current study revealed that knowledge, attitude, and perception were significantly associated with the uptake of the COVID‐19 vaccine. The results indicated that participants with good knowledge were two times more likely to uptake the COVID‐19 vaccine than those with poor knowledge. This result also was observed in previous studies [14, 44]. Thus, improving population knowledge about COVID‐19 vaccines might be a way forward to maximize the vaccination uptake. Therefore, health education on vaccine safety, side effects, and its effectiveness must be regularly provided to the population to improve their level of knowledge.

In addition, the current study found that a good attitude and positive perception toward COVID‐19 vaccines was strongly associated with vaccine uptake, consistent with findings from previous studies [25, 45]. This association may be explained by the fact that attitudes and perceptions play important roles in influencing individuals' willingness to utilize healthcare services. These factors are often shaped by the community's overall satisfaction with the quality of health services provided such as the availability of medicines, respectful treatment by healthcare workers, waiting times, and accessibility of services [46]. When people have positive experiences within the health system, they are more likely to develop trust and confidence in health recommendations, including vaccines. Conversely, negative experiences can lead to skepticism, reluctance, or rejection of new interventions. As a result, people may hesitate to accept newly introduced drugs or vaccines, especially when trust in the health system is lacking. For achieving universal uptake of COVID‐19 vaccination, it is essential that all stakeholders understand and address community attitudes and perceptions, which are key to designing effective and culturally sensitive interventions.

To the best of our knowledge, this is the first study in Tanzania to assess socioeconomic inequalities in the uptake of COVID‐19 among adults in the study area. The use of a big sample size which included participants from major cities across all zones of Tanzania ensures the representativeness of the collected data. This implies a high degree of generalizability and accuracy in characterizing the situation of socioeconomic inequalities in the uptake of the COVID‐19 vaccine in Tanzania. Nevertheless, the cross‐sectional nature of the study is a limitation, and the study failed to document whether the exposures occurred before the outcome and this might have affected the observed association. Therefore, the present findings should be interpreted with caution. Additionally, the use of education as a proxy for SES may not fully capture the multidimensional nature of SES in Tanzania. Although education is often used as a practical and stable indicator, it may not accurately reflect income levels, employment type, or access to health‐related resources, particularly in rural and informal economies. In Tanzania, individuals with similar education levels may experience vastly different economic conditions due to factors such as regional disparities, employment opportunities, or family responsibilities. As such, relying solely on education could have led to some misclassification in assessing the true socioeconomic gradient in vaccine uptake. Therefore, future studies may benefit from including multiple SES indicators such as household income, asset ownership, or occupation to provide a more comprehensive understanding.

## 5 Conclusions and Future Implications

The current study is the first to investigate the regional variation of socioeconomic inequalities in the uptake of COVID‐19 vaccines in a context with high hesitancy. The results of this study provided evidence that socioeconomic inequalities in the uptake of COVID‐19 vaccine favor educated individuals. Therefore, the study underscores the importance of education in reducing or eliminating inequalities in the uptake of COVID‐19 vaccines.

Based on the study findings, we recommend strengthening health education campaigns targeting individuals with low or no formal education through culturally appropriate messaging in local languages to improve knowledge and perceptions about COVID‐19 vaccines. Health authorities should also enhance communication strategies by involving trusted community figures to counter misinformation and vaccine hesitancy. To promote equity, outreach programs should prioritize socioeconomically disadvantaged groups, including those in informal employment. Integrating vaccination services into routine healthcare and continuously monitoring public perceptions will further support efforts to increase vaccine uptake and build trust in the health system.

## Author Contributions

**Deogratius Bintabara:** conceptualization, investigation, writing – original draft, methodology, writing – review and editing, software, formal analysis, project administration, supervision, data curation. **Gabriel Mchonde:** conceptualization, investigation, visualization, writing – review and editing, validation, supervision, writing – original draft.

## Disclosure

All authors have read and approved the final version of the manuscript. The corresponding author had full access to all of the data in this study and takes complete responsibility for the integrity of the data and the accuracy of the data analysis.

## Conflicts of Interest

The authors declare no conflicts of interest.

## Transparency Statement

The lead author Deogratius Bintabara affirms that this manuscript is an honest, accurate, and transparent account of the study being reported; that no important aspects of the study have been omitted; and that any discrepancies from the study as planned (and, if relevant, registered) have been explained.

## Data Availability

The data set used for this study is restricted by the Ethical Research Committee of The University of Dodoma, as it contains sensitive patient information. However, it can be accessed upon reasonable request from the Directorate of Research Publication and Consultancy, The University of Dodoma, P.O. Box 259, Dodoma, Tanzania (drpc@udom.ac.tz).
